# Neuroimaging in Parkinson’s disease dementia: connecting the dots

**DOI:** 10.1093/braincomms/fcz006

**Published:** 2019-07-08

**Authors:** Rimona S Weil, Joey K Hsu, Ryan R Darby, Louis Soussand, Michael D Fox

**Affiliations:** 1 Dementia Research Centre, UCL, London; 2 Wellcome Centre for Human Neuroimaging, UCL, London; 3 Berenson-Allen Center, Beth Israel Deaconess Medical Center, Harvard Medical Center, Boston, MA, USA; 4 Department of Neurology, Vanderbilt University Medical Center, Nashville, TN, USA; 5 Department of Neurology, Massachusetts General Hospital, Harvard Medical School, Boston, MA, USA; 6 Athinoula A. Martinos Center for Biomedical Imaging, Massachusetts General Hospital, Harvard Medical School, Charlestown, MA, USA

**Keywords:** Parkinson’s disease, dementia, mild cognitive impairment, visual hallucinations, imaging

## Abstract

Dementia is a common and devastating symptom of Parkinson’s disease but the anatomical substrate remains unclear. Some evidence points towards hippocampal involvement but neuroimaging abnormalities have been reported throughout the brain and are largely inconsistent across studies. Here, we test whether these disparate neuroimaging findings for Parkinson’s disease dementia localize to a common brain network. We used a literature search to identify studies reporting neuroimaging correlates of Parkinson’s dementia (11 studies, 385 patients). We restricted our search to studies of brain atrophy and hypometabolism that compared Parkinson’s patients with dementia to those without cognitive involvement. We used a standard coordinate-based activation likelihood estimation meta-analysis to assess for consistency in the neuroimaging findings. We then used a new approach, coordinate-based network mapping, to test whether neuroimaging findings localized to a common brain network. This approach uses resting-state functional connectivity from a large cohort of normative subjects (*n* = 1000) to identify the network of regions connected to a reported neuroimaging coordinate. Activation likelihood estimation meta-analysis failed to identify any brain regions consistently associated with Parkinson’s dementia, showing major heterogeneity across studies. In contrast, coordinate-based network mapping found that these heterogeneous neuroimaging findings localized to a specific brain network centred on the hippocampus. Next, we tested whether this network showed symptom specificity and stage specificity by performing two further analyses. We tested symptom specificity by examining studies of Parkinson’s hallucinations (9 studies, 402 patients) that are frequently co-morbid with Parkinson’s dementia. We tested for stage specificity by using studies of mild cognitive impairment in Parkinson’s disease (15 studies, 844 patients). Coordinate-based network mapping revealed that correlates of visual hallucinations fell within a network centred on bilateral lateral geniculate nucleus and correlates of mild cognitive impairment in Parkinson’s disease fell within a network centred on posterior default mode network. In both cases, the identified networks were distinct from the hippocampal network of Parkinson’s dementia. Our results link heterogeneous neuroimaging findings in Parkinson’s dementia to a common network centred on the hippocampus. This finding was symptom and stage-specific, with implications for understanding Parkinson’s dementia and heterogeneity of neuroimaging findings in general.

## Teaser

Heterogeneous neuroimaging findings in Parkinson’s dementia localise to a common brain network centred on the hippocampus. This result is symptom and disease-stage specific, as visual hallucinations and mild cognitive impairment map to different networks. These results make sense of an otherwise discordant neuroimaging literature, lending insight into Parkinson’s dementia.

## Graphical Abstract

**Graphical Abstract:**
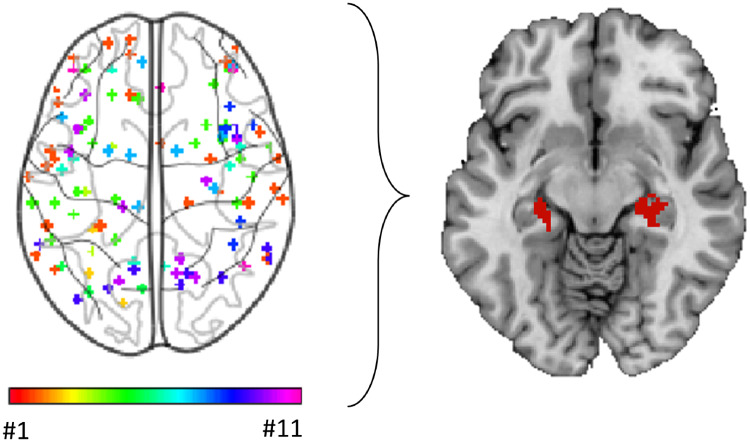

## Introduction

Dementia is a common and debilitating aspect of Parkinson’s disease: 50% of patients will develop dementia within 10 years of diagnosis (Williams-Gray *et al.*, 2013), and it carries significant societal and economic burden (Spottke *et al.*, 2005; Leroi *et al.*, 2012) with high levels of frailty and nursing home admissions (Fredericks *et al.*, 2017; Weir *et al.*, 2018). Identifying the neuroanatomical substrate of Parkinson’s disease with dementia (PD dementia) could aid prognosis and treatment development. Unfortunately, this neuroanatomical substrate remains unclear.

One possibility is that PD dementia stems from the hippocampus, a region known to play a critical role in memory and in other forms of dementia (Fox *et al.*, 1996; Seeley *et al.*, 2009; Darby *et al.*, 2019). Memory problems are frequently the first subjective cognitive complaint in Parkinson’s disease (Noe *et al.*, 2004) and are a prominent component of PD dementia (Whittington *et al.*, 2000; Bronnick *et al.*, 2007; Muslimovic *et al.*, 2007; Reid *et al.*, 2011; Wang *et al.*, 2015), forming part of the diagnostic criteria for PD dementia (Emre *et al.*, 2007). In patients with PD dementia the hippocampus shows a higher density of Lewy pathology (Harding and Halliday, 2001; Apaydin *et al.*, 2002; Arnold *et al.*, 2013; Hall *et al.*, 2014), reduction in cholinergic activity (Hall *et al.*, 2014) and progressive atrophy with disease progression (Aybek *et al.*, 2009; Weintraub *et al.*, 2011, 2012; Morales *et al.*, 2013; Kandiah *et al.*, 2014; Mak *et al.*, 2015; Gee *et al.*, 2017; Mihaescu *et al.*, 2018).

However, the role of the hippocampus in PD dementia remains uncertain for several reasons. First, although memory problems are an early subjective complaint (Noe *et al.*, 2004), objective testing usually shows early deficits in visuospatial and executive function (Janvin *et al.*, 2006*a*; Williams-Gray *et al.*, 2013; Kalbe *et al.*, 2016). At this stage, patients are often considered as having PD with mild cognitive impairment (PD-MCI) (Emre *et al.*, 2007; Kehagia *et al.*, 2013). Although 90% of these patients will eventually progress to PD dementia, worse visuospatial deficits, not memory deficits, are associated with rapid progression (Williams-Gray *et al.*, 2013; Weil *et al.*, 2017). Second, most PD dementia patients have co-morbid symptoms such as visual hallucinations, whose neural substrate is also unclear but is unlikely to localize to the hippocampus (Fenelon *et al.*, 2000; Gallagher *et al.*, 2011). Finally, PD dementia is associated with Lewy pathology and atrophy throughout nearly the entire brain (Hurtig *et al.*, 2000; Braak *et al.*, 2005; Irwin *et al.*, 2012). Neuroimaging studies of PD dementia have been particularly heterogeneous (Lanskey *et al.*, 2018), with atrophy or hypometabolism reported in frontal (Song *et al.*, 2011; Melzer *et al.*, 2012), temporal (Melzer *et al.*, 2012; Pagonabarraga *et al.*, 2013), parietal (Melzer *et al.*, 2012; Pereira *et al.*, 2014), occipital (Melzer *et al.*, 2012) and insular cortices (Mak *et al.*, 2014) as well as numerous subcortical regions (Melzer *et al.*, 2012; Foo *et al.*, 2017; Schneider *et al.*, 2017). Different meta-analyses of the coordinates reported by these studies have also been inconsistent (Minkova *et al.*, 2017; Mihaescu *et al.*, 2018).

An assumption underlying many conventional neuroimaging studies is that abnormalities should localize to specific brain regions in order to explain specific symptoms (Eickhoff *et al.*, 2009). However, some symptoms may localize better to brain networks, rather than specific brain regions (Fox *et al.*, 2005; Dickerson and Sperling, 2009; Seeley *et al.*, 2009). We have used this approach to link lesions found in disparate brain regions that produce similar symptoms to a common brain network, a technique known as lesion network mapping (Boes *et al.*, 2015; Fox, 2018; Joutsa *et al.*, 2018*a*). Recently, we validated an extension of lesion network mapping termed coordinate-based network mapping (Darby *et al.*, 2019). We showed that heterogeneous neuroimaging findings in Alzheimer’s disease map to a common brain network, centred on the hippocampus (Darby *et al.*, 2019). This result was specific compared to neurodegenerative diseases that are not characterized by memory decline.

Here, we apply this technique to PD dementia. We hypothesize that: (i) coordinate network mapping will reveal a common network involved in PD dementia centred on the hippocampus; (ii) this network will be specific compared with the highly co-morbid symptom of visual hallucinations; (iii) this network will be specific compared to PD-MCI which is an earlier stage of PD dementia more commonly characterized by visuospatial or executive dysfunction.

## Materials and methods

### Search strategy

We identified studies reporting neuroimaging abnormalities in patients with Parkinson’s disease dementia and with mild cognitive impairment by performing a search of the PubMed databases for papers published between 1 January 1985 and 4 June 2018. Four sets of keywords were used: Parkinson or Parkinson’s; dement*, dementia, mild cognitive impairment or MCI; MRI or magnetic resonance imaging combined with voxel-based morphometry, VBM or struct*; and PET, fludeoxyglucose (FDG)-PET or single photon emission computed tomography (SPECT), restricted to human studies. A similar search was performed to identify relevant studies on visual hallucinations in Parkinson’s disease and included hallucinations, Parkinson’s disease, MRI and FDG-PET or SPECT, as above. The reference lists of relevant review articles were then hand searched for potential missed studies.

### Inclusion and exclusion criteria

**Figure 1:**
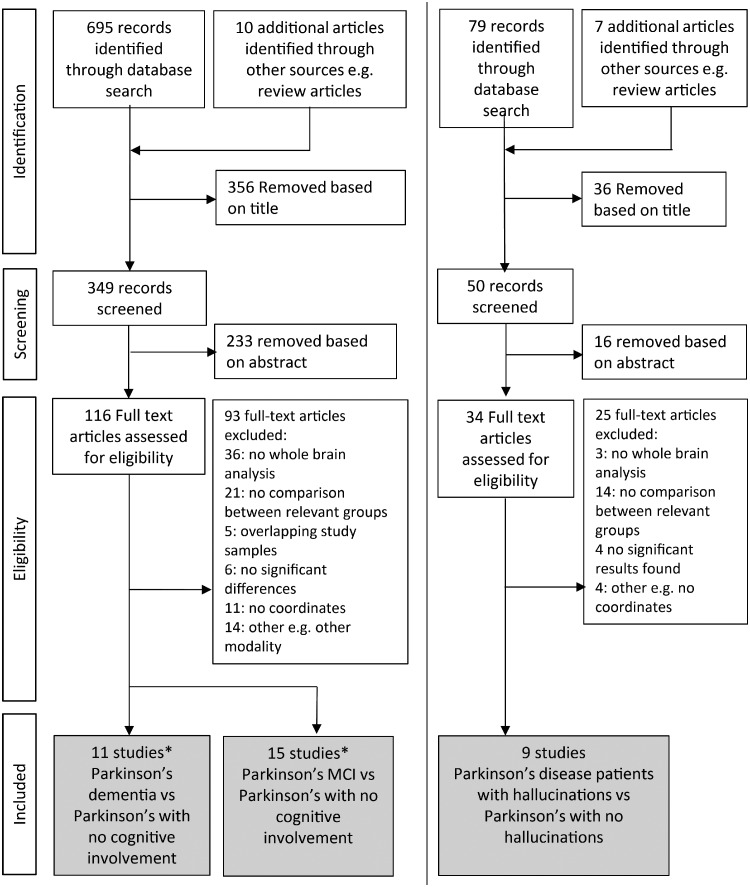
**Systematic literature search and study selection.** Neuroimaging studies of Parkinson’s disease (PD) dementia, PD with visual hallucinations and PD-MCI were selected in accordance with PRISMA guidelines. *Three studies included both PD dementia and PD-MCI comparisons.

The meta-analyses included only articles that (i) involved patients with Parkinson’s disease and dementia (or hallucinations), with PD dementia defined as a dementia syndrome that developed in the context of established Parkinson’s disease (Emre *et al.*, 2007); (ii) reported coordinates for atrophy (using VBM or cortical thickness measures) or hypometabolism (FDG-PET or SPECT) between the relevant patient groups; (iii) used comparisons between the symptom in question and Parkinson’s patients without that symptom; (iv) reported whole-brain results for these changes; (v) coordinates were reported in stereotactic space (montreal neurological institute (MNI) or Talairach). We excluded the following: (i) studies exclusively reporting changes in dementia with Lewy Bodies; (ii) studies without direct comparisons between patient groups (e.g. brain regions correlating with cognitive scores); (iii) non-original or duplicate studies; (iv) studies that confined their search within specific regions of interest; (v) studies that reported no differences between patient groups; (vi) case reports; (vii) studies that did not report coordinates or where reported coordinates diverged significantly from reported locations. (See Fig. 1 for flow diagrams for the searches and Tables 1–3 for included studies for each of the searches.)

**Table 1:** Clinical characteristics and scanning modalities of studies of Parkinson’s dementia (PDD) versus Parkinson’s without cognitive involvement (PD)

First author	Modality	*N*	*N*	Age	Age PD	MMSE PDD	MMSE PD	H&Y PDD	H&Y PD	UPDRS PDD	UPDRS PD
		PDD	PD	PDD							
		Total *n* = 175	Total *n* = 210								
(1) Beyer^b^^,^^c^	VBM	16	20	73.5	72.5	19.4	28.2	3	2.4		
(2) Burton^b^	VBM	26	31	72.3	75.2	18.9	26.4			36.4	25.8
(3) Gee^c^	VBM	23	10	71.6	69.4	27.3	28.9			14.4	15.3
(4) Goldman^b^	VBM	24	26								
(5) Klein^c^	FDG-PET	8	9	62	67	21	28.4	2	3	24	25
(6) Lee^b^	VBM	16	16	69.9	68.3	19.6	27.3	2.6	1.7		
(7) Nagano-Saito^b^^,^^c^	VBM	9	17	67.3	65.4	16.1	27.9	3.3	3.1		
(8) Song^b^	VBM	18	23	72	69.1	18.1	28.6			32.1	16.9
(9) Tang^b^^,^^c^	FDG-PET	10	30	61.4	61.9	23.2	28.5	2.5	1.8	30.7	23
(10) Xia	VBM	12	12	69.3	65.6	23.4	28.1	3	1.8	44	14.3
(11) Yong^b^	FDG-PET	13	16	73.4	64.2	15.4	27.3	3.2	2.1		
Summary (mean(SD))		16 (6)	19 (8)	69.3 (4)	67.8 (4)	20.2 (4)^a^	28.0 (0.8)^a^	2.8 (0.5)	2.3 (0.6)	30.3 (10)	20.1 (5)

a Wilcox test shows significant difference between groups (Other comparisons are not significantly different).

b Indicates established criteria were used to define PD dementia. Extended neuropsychological testing was used in the remaining studies.

c Indicates the study matched PDD and PD groups for motor stage.

FDG-PET, fluorodeoxyglucose positron emission tomography; H&Y, Hoehn and Yahr; MMSE, mini-mental state examination; PDD, Parkinson’s disease dementia; SPECT, single photon emission computed tomography; UPDRS, Unified Parkinson’s disease rating scale score (part III, motor); VBM, voxel-based morphometry.

**Table 2:** Clinical characteristics and scanning modalities of included studies of Parkinson’s hallucinations

First author	Modality	*N* PDVH	*N* PD	Age PDVH	Age PD	MMSE PDVH	MMSE PD	H&Y PDVH	H&Y PD	UPDRS PDVH	UPDRS PD
		Total *n* = 168	Total *n* = 234								
(27) Boecker^b^	FDG-PET	8	11	72.88	70.56	25.75	26.82			46.25	32.73
(28) Gasca-Salas^b^	FDG-PET	9	12	70.7	70.8	27	25.9			16.1	17
(29) Goldman^b^	VBM	25	25	75.4	74.8	25.1	23.9	3	3	43.5	39
(30) Lee^b^	VBM	10	21	69.4	66.2	27.6	28.2	2.2	1.8	22.5	16.4
(31) Oishi	SPECT	24	41	69.5	68.6	25.1	26.5	3.3	3		
(32) Pagonobarraga^b^	VBM	15	27	64.1	66.3	135^a^	136^a^	1.9	1.9	21.7	18.6
(33) Ramirez-Ruis	VBM	18	20			27	29.1	3.2	2.5	29.3	24.5
(34) Shin^b^	VBM	46	64	71.3	70.7	25.2	25.7			24.1	21.6
(35) Watanabe^b^	VBM	13	13	66.6	63.6	27.9	90	2.9	2.4	23.4	28.6
Summary (mean (SD))		19 (12)	26 (17)	70.0 (4)	68.9 (3.5)	26.3 (1)	26.9 (2)	2.8 (0.6)	2.4 (0.5)	28.8 (11)	24.8 (8)

a Matis dementia rating supplied.

b Indicates established criteria were used to define PD hallucinations.

FDG-PET, fluorodeoxyglucose positron emission tomography; MMSE, mini-mental state examination; PDVH, Parkinson’s disease with visual hallucinations; SPECT, single photon emission computed tomography; UPDRS, unified parkinson's disease rating scale score (part III, motor); VBM, voxel-based morphometry.

No significant differences between groups for any of these comparisons.

**Table 3:** Clinical characteristics and scanning modalities of included studies of PDMCI versus Parkinson's disease with no cognitive involvement

First author	Modality	*N* PD-MCI	*N* PD	Age PD-MCI	Age PD	MMSE PD-MCI	MMSE PD	H&Y PD-MCI	H&Y PD	UPDRS PD-MCI	UPDRS PD
		Total *n* = 355	Total *n* = 489								
(12) Beyer^c^	VBM	8	12	77.4	69	25.9	29.4	2.6	2.3		
(13) Danti^a^	Freesurfer	18	18	66.5	60.6	26.4	28.7	1.6	1.3		
(14) Garcia-Garcia^a^	FDG-PET	28	21	71.5	67	28	29.5	2.9	2.6	17.7	16.4
(15) Hosokai^d^	FDG-PET	13	27	67.6	65.7	27.1	27.9	2.7	2.5	22.4	18.5
(16) Huang^c^	FDG-PET	18	18	62.4	59	27.1	28.2	3.6	3.1	34.9	29.2
(17) Lyoo^c^	FDG-PET	18	20	65.5	62	27	29	2.3	2.3	25.5	22
(18) Mak^a^	VBM	24	66	68.99	63.48	26.91	28.36	1.81	1.91	19.96	17.44
(19) Mak^a^	Cortical thickness	39	66	69.4	62.9	28.1	29.1	2.1	1.9	29	25.3
(20) Nobili^c^	SPECT	15	15	71.5	70.8	27.3	28.7			22.9	15.3
(21) Pagonobarraga^d^	Freesurfer	26	26	73.3	71.5	128^e^	134^e^	2	2.2	21	24
(22) Pereira^b^	Freesurfer	33	90	63.4	59.4	25.7^f^	28.1^f^	2	2	21.5	19.6
(23) Segura^a^	Freesurfer	47	43	67.72	60.77	28.68	29.47			17.79	13.16
(24) Song^c^	VBM	27	23	71.3	69.1	25.8	28.6			18.6	16.9
(25) Tang^a^	FDG-PET	20	30	61.9	61.9	28.4	28.5	2.1	1.8	30	23
(26) Zhang^c^	VBM	21	14	63.8	58.5	28.85	29.07	1.77	1.42		
Summary (mean (SD))		24 (10)	33 (23)	68.1 (4)*	64.1 (4)*	27.3 (1)*	28.8 (0.5)*	2.3 (0.6)	2.1 (0.5)	23.4 (5)	20.1 (5)

* Significant difference between groups. (Other comparisons are not significantly different).

a Established criteria used to define PD-MCI (Litvan *et al.*, 2012).

b Close approximation of established criteria used to define PD-MCI.

c Previous criteria used to define PD-MCI (Petersen *et al.*, 2001).

d Alternative method used to define PD-MCI (Clinical Dementia Rating score of 0.5).

e Matis dementia rating supplied.

f MOCA supplied.

FDG-PET, fluorodeoxyglucose positron emission tomography; H&Y, Hoehn and Yahr; MMSE, mini-mental state examination; PD-MCI, Parkinson’s disease with mild cognitive impairment; SPECT, single photon emission computed tomography; UPDRS, Unified Parkinson’s disease rating scale score (part III, motor); VBM, voxel-based morphometry.

### Data extraction and demographics

Data were extracted from each of the identified studies using a predefined data extraction form, to include information on author, publication year, sample size, demographics, clinical information, modality and coordinates. Talairach coordinates were converted into MNI coordinates using the automated transformation provided with GingerALE (http://www.brainmap.org/ale/), unless the study specified that the original analysis was conducted in MNI space and converted *post hoc* into Talairach space, in which case we used the conversion provided by MNI to Talairach converter programme (http://sprout022.sprout.yale.edu/mni2tal/mni2tal.html).

We tested for significant demographic differences such as age and Hoehn and Yahr using two-tailed Welch’s *t*-tests or Mann-Whitney-Wilcoxon tests for non-normally distributed data. *P *<* *0.05 was accepted as threshold for statistical significance. Analyses were performed in R (https://www.r-project.org/).

### Activation likelihood estimation meta-analyses

We used GingerALE 2.3.6 (http://brainmap.org/ale) to perform an activation likelihood estimation meta-analysis for Parkinson’s dementia compared with Parkinson’s without cognitive involvement using standard methods (Eickhoff *et al.*, 2009, 2012). In brief, a 3D Gaussian probability distribution is generated centred on each individual study coordinate, and modified by the sample size from each study. This enabled us to estimate the uncertainty surrounding each coordinate. These distributions were then combined across all the studies for the relevant comparison to produce activation likelihood estimate maps. We used the threshold of *P *<* *0.05 false discovery rate (FDR)-corrected to determine significance and also tested convergence against a null distribution of 1000 simulated datasets with identical numbers of foci experiments and subjects with the foci randomly distributed. For these meta-analyses, cluster-forming threshold was set at *P *<* *0.001 and cluster-level inference threshold at *P *<* *0.05. The same approach was used to perform separate activation likelihood estimation meta-analyses for Parkinson’s with and without visual hallucinations; and Parkinson’s-MCI (PD-MCI) compared with Parkinson’s without cognitive involvement. We also directly compared studies of PD dementia to those of PD hallucinations and PD-MCI, using the same statistical methods described above.

### Coordinate-based network mapping

Next, we used a recently validated technique termed coordinate-based network mapping (Darby *et al.*, 2019) to test the hypothesis that neuroimaging findings from studies of PD dementia would localize to a common brain network. This technique is modified from lesion network mapping, a technique used to test whether brain lesions map to a common brain network (Darby *et al.*, 2017; Horn *et al.*, 2017). For each neuroimaging study, we created 4 mm spherical seeds at the reported coordinates. We added these seeds together to produce one combined seed for each study (Eickhoff *et al.*, 2009; Yarkoni *et al.*, 2011; Darby *et al.*, 2019). We then identified the network of brain regions functionally connected to the seed using a connectome database from 1000 normal subjects (Yeo *et al.*, 2011; Holmes *et al.*, 2015). We thresholded each connectivity map at t ≥ 7 (corresponding to family-wise error (FWE) voxel-based correction *P *<* *10^−6^) (Joutsa *et al.*, 2018*b*) to derive a network map for each study. These binarized maps were then overlapped to identify network connections common to the greatest number of studies of PD dementia. We performed this analysis across the entire brain, as well as for an a priori region of interest (ROI) in the hippocampus, defined using the publically available SPM anatomical toolbox (http://www.fz-juelich.de/inm/inm-1/DE/Forschung/_docs/SPMAnatomyToolbox/SPMAnatomyToolbox_node.html) (Amunts *et al.*, 2005). We also computed functional connectivity between each study’s coordinates and this a priori hippocampal ROI using our 1000 subject normative connectome. Pearson’s correlations coefficients were converted to a normal distribution using Fisher’s r to z transform then averaged across our 1000 subjects. We tested for significance of this connection across studies using permutation testing in R (one-sample, two tailed, *P *<* *0.05). We used one-tailed significance testing for this ROI analysis given our a priori hypothesis that coordinates from PD dementia studies should be positively connected to the hippocampus.

### Specificity of network localization for Parkinson’s disease dementia

To test for symptom specificity, we repeated the above analyses using studies of PD visual hallucinations, defined as the presence of visual hallucinations in the context of Parkinson’s disease, where patients with visual hallucinations were directly compared with patients with PD without hallucinations. To test for stage specificity, we repeated the above analyses using studies of PD-MCI. PD-MCI was defined as cognitive deficits in the context of established Parkinson’s disease not of sufficient severity to impair functional independence (Petersen *et al.*, 2001; Winblad *et al.*, 2004; Litvan *et al.*, 2012). Studies were selected that directly compared patients with PD-MCI with Parkinson’s and no cognitive involvement.

Network connectivity maps from studies of PD hallucinations or studies of PD-MCI were statistically compared to network maps from studies of PD dementia on a voxel-wise basis using permutation testing within FSL PALM (two-tailed, voxel-based FWE correction *P *<* *0.05). Permutation testing with voxel-based FWE correction for multiple comparisons reduces the risk of false positives (Eklund *et al.*, 2016) and is consistent with best-practice recommendations for neuroimaging (Poldrack *et al.*, 2017). To maximize sensitivity, this voxel-wise analysis was restricted to a mask defined by our a priori hippocampal ROI. Functional connectivity between study coordinates and our a priori hippocampal ROI was also computed and compared using permutation testing within R (https://www.r-project.org/) (two sample, one tailed, *P *<* *0.05). We used one-tailed significance testing for this ROI analysis given our a priori hypothesis that coordinates from PD dementia studies should be more connected to the hippocampus than studies of PD visual hallucinations or studies of PD-MCI.

In a *post hoc* analysis, we also tested for specificity of our PD visual hallucination findings to the lateral geniculate nucleus (LGN). For this analysis, an LGN ROI was generated using 18 mm spheres centred on previously described coordinates (Burgel *et al.*, 2006). Note that unlike our hippocampus ROI, our LGN ROI was not specified a priori, but selected *post hoc* based on the results of our whole-brain network mapping of PD visual hallucinations.

### Data availability

The data on which this study is based were all obtained from published and publically available reports (see Tables 1–3 for details).

## Results

### Heterogeneous neuroimaging findings for Parkinson’s dementia are linked to a common network centred in the hippocampus

We identified 11 studies that reported neuroimaging abnormalities in patients with PD dementia (total *n* = 175) compared to PD without cognitive impairment (total *n* = 210, Table 1). All studies used established criteria to diagnose Parkinson’s diease (Calne *et al.*, 1992; Hughes *et al.*, 1992; Larsen *et al.*, 1994), and the majority (8 out of 11) used established criteria to define PD dementia (American Psychiatric Association, 1996; Emre *et al.*, 2007). Between groups, there was no significant difference in age (t(18) = 0.8, *P *=* *0.46), disease stage (H&Y, t(11) = −1.9, *P *=* *0.085), or motor function (unified Parkinson's disease rating scale (UPDRS) III, t(−2.2) = 7, *P *=* *0.063), but a large difference in cognition as expected [mini-mental state examination, W = 98, *P *=* *0.00032]. Cognitive scores in the PD dementia group were similar across studies (mean mini-mental state examination 20.2, SD 3.6).

**Figure 2:**
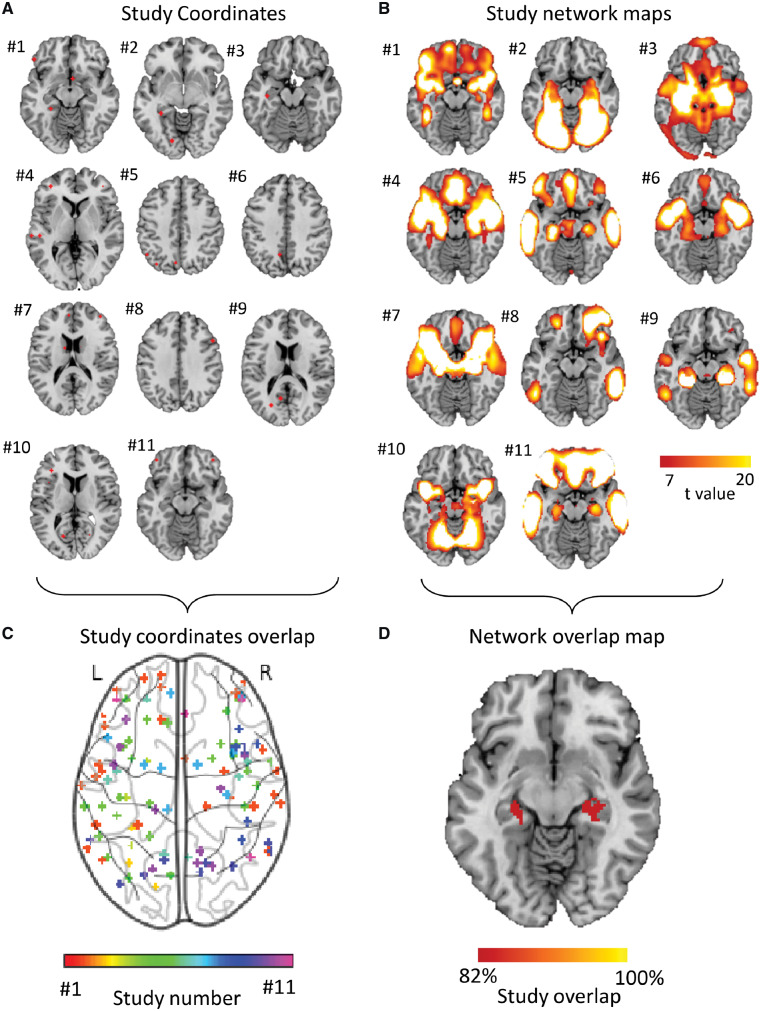
**Heterogeneous neuroimaging findings in Parkinson’s disease dementia are part of a common brain network centred on the hippocampus.** (**A**) Study coordinates. Location of coordinates for each study of Parkinson’s dementia compared with Parkinson’s without cognitive involvement. Spherical seeds were generated at each reported significant coordinate for each study of PD dementia, then added together to create one map of neuroimaging findings for each study. Numbers refer to the study number as listed in Table 1. (**B**) Study network maps. Regions significantly connected to each study’s neuroimaging findings were calculated using a large (*n* = 1000) normative connectome, creating a network map for each study (FWE-corrected *P* < 10^−6^). Locations of network connectivity for each study of Parkinson’s dementia compared with Parkinson’s without cognitive involvement. (**C**) Study coordinates overlap. Combined location of all coordinates across all studies of PD dementia shows pronounced heterogeneity. Each study is represented by a different colour. (**D**) Network overlap map. Network maps from each study were overlaid to identify functional connections common to the greatest number of studies in a whole-brain analysis. Over 80% of studies were functionally connected to the bilateral hippocampus. Section at *z* = −16 is shown.

Neuroimaging findings from these studies were highly heterogeneous (Fig. 2A and C). Using standard meta-analytic methods, no voxels or clusters appeared more often than expected by chance. Only 4/11 studies (36%) contributed to the most consistent finding, which was in the right insula (MNI coordinates 41.5, 6.8, −17.7).

Next, we tested whether these heterogeneous neuroimaging findings localized to a common brain network. For each study, we generated a 4-mm sphere at each reported coordinate to obtain a study-specific map of abnormalities related to Parkinson’s dementia. We then identified the network of brain regions functionally connected to each study-specific map using a large (*n* = 1000) normative connectome. Each network map was thresholded (*t* ≥ 7, FWE *P *<* *10^−6^), binarized, then overlapped to identify regions common to all or most studies (Fig. 2D). Applying this approach to the 11 studies of PD dementia in a whole-brain analysis, we found over 90% reproducibility, with peak network overlap in the right hippocampus (Fig. 2D). The second highest peak was in the left hippocampus (>80% of studies). Both areas of peak overlap fell within our a priori hippocampal ROI, and connectivity to this ROI was significant across studies of PD dementia (*t* = 3.0, *P *=* *0.01). In summary, although neuroimaging studies of atrophy and hypometabolism in PD dementia reported heterogeneous coordinates, these coordinates were part of a common brain network centred on the hippocampus.

### Specificity of network localization compared to visual hallucinations in Parkinson’s disease

To determine whether network localization to the hippocampus was specific to the symptom of dementia in PD, we performed a separate meta-analysis of hallucinations. We identified nine studies reporting atrophy or hypometabolism in patients with PD hallucinations (total *n* = 168) compared with PD without hallucinations (total *n* = 234, Table 2). All studies used UK Brain Bank criteria to define Parkinson’s disease (Hughes *et al.*, 1992) and 7 of the 9 studies used established methods to define the presence of visual hallucinations (Cummings, 1997; Ravina *et al.*, 2007; Goetz *et al.*, 2008). All except two studies (Ramirez-Ruiz *et al.*, 2007; Lee *et al.*, 2017) controlled for cognition. Between groups there was no significant difference in age (t(14) = 0.59, *P *=* *0.56), disease stage (H&Y, t(10) = 1.0, *P *=* *0.34), or cognition (mini-mental state examination, t(12) = −0.7, *P *=* *0.48).

**Figure 3:**
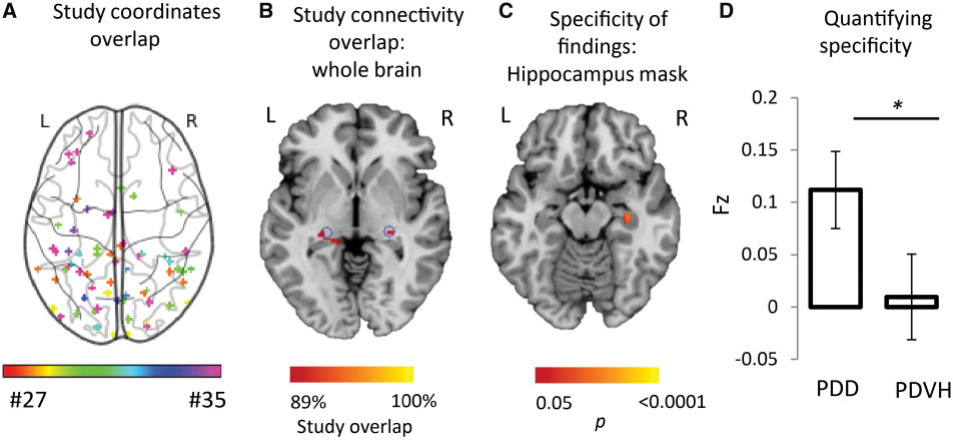
**Heterogeneous neuroimaging findings in Parkinson’s disease hallucinations are part of a different brain network than PD dementia, centred on the lateral geniculate nucleus.** (**A**) Combined location of all coordinates across all studies of Parkinson’s with visual hallucinations shows pronounced heterogeneity. Each study is represented by a different colour. (**B**) Connectivity maps (across the whole brain) for each study of PD hallucinations were generated and overlaid, showing network overlap in the lateral geniculate nuclei bilaterally. Section shown is at *z* = −4. Blue circles indicate location of lateral geniculate nucleus based on published coordinates (Burgel *et al.*, 2006). (**C**) Direct comparison of network maps generated from studies of PD dementia and PD hallucinations shows specificity of hippocampal connectivity to studies of PD dementia. Map is masked to the hippocampi and FWE-corrected *P* < 0.05. Section shown is at *z* = −16. (**D**) Connectivity to our a priori ROI in the hippocampus was significantly stronger for studies of PD dementia compared to studies of PD hallucinations. Coordinate and network maps for all studies can be viewed in Fig. 5. * *P* < 0.05; PDD, Parkinson’s disease dementia; PDVH, Parkinson’s disease with visual hallucinations.

We found that using standard methods for meta-analysis, neuroimaging results were heterogeneous (Fig. 3A). No consistent clusters were found, either across studies of PD hallucinations or when comparing studies of PD hallucinations with studies of PD dementia.

When we applied the same coordinate network mapping approach we used for dementia to studies of PD hallucinations, we again found that the vast majority of studies (89%) mapped to a common brain network. However, this time the peak network overlap was the lateral geniculate nuclei (LGN) in the thalamus, not the hippocampus (Fig. 3B). Directly testing for specificity of hippocampal connectivity for PD dementia, we found that coordinates from studies of PD dementia were more connected to voxels in the right hippocampus (Fig. 3C, *P *<* *0.05 FWE-corrected) and more connected to our a priori hippocampal ROI (*z* = −1.75, *P *=* *0.04, one-tailed permutation test, Fig. 3D) compared to studies of PD hallucinations.

A *post hoc* analysis tests for specificity of LGN connectivity for PD hallucinations (versus studies of PD dementia) found that coordinates from studies of PD hallucinations were more connected to voxels in the right LGN (*P *<* *0.05 FWE-corrected) and to an anatomically defined ROI in bilateral LGN (*z* = 1.91, *P *=* *0.028, one-tailed permutation test).

### Network localization reveals separate networks involved at milder stages of cognitive involvement in Parkinson’s disease

Next, we examined whether network localization would reveal separate networks according to *stage* of cognitive involvement in Parkinson’s disease. We identified 15 studies examining differences in atrophy or hypometabolism between people with PD-MCI (total *n* = 355) and those with Parkinson’s disease and no cognitive impairment (total *n* = 489, Table 3). Thirteen out of 15 studies used established criteria for Parkinson’s disease diagnosis (Hughes *et al.*, 1992; Larsen *et al.*, 1994; Gelb *et al.*, 1999) and 13 out of 15 studies used recent (Litvan *et al.*, 2012) or previous (Petersen *et al.*, 2001) criteria for PD-MCI. Despite these different methods, there was relatively little variability in mini-mental state examination scores across the studies (PD-MCI 27.3 (SD 1.0), Parkinson’s disease without cognitive involvement 28.8 (SD 0.5)). Between groups, PD-MCI patients were older (t(28) = 2.5, *P *=* *0.018)) and showed worse cognition (t(18)=4.7, *P *<* *0.001), but did not differ in terms of disease stage (H&Y, t(22) = 0.8, *P *=* *0.42) or motor disability (UPDRS III, t(22) = 1.6, *P *=* *0.12; Table 3).

**Figure 4:**
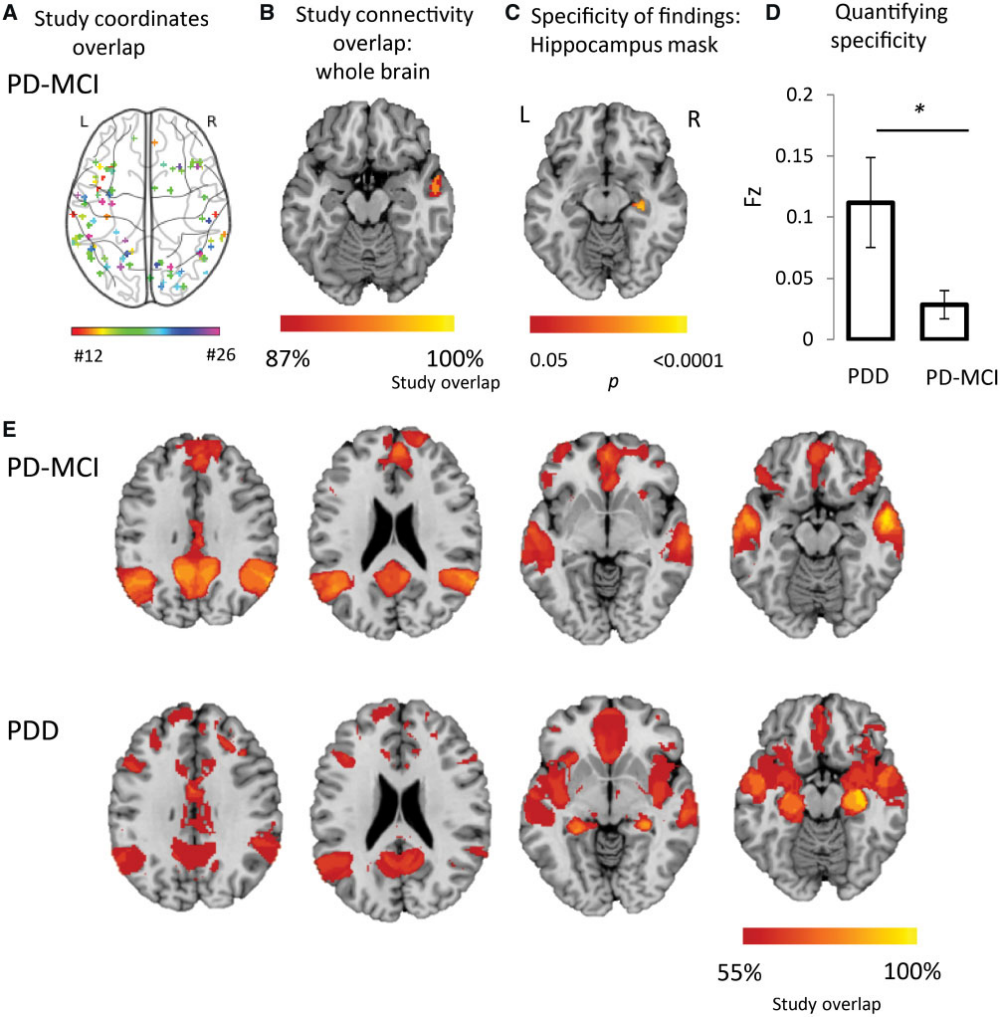
**Heterogeneous neuroimaging findings in Parkinson’s disease MCI are part of a network centred on posterior nodes of the default mode network.** (**A**) Combined location of all coordinates across all studies of Parkinson’s mild cognitive impairment (PD-MCI) shows pronounced heterogeneity. Each study is represented by a different colour. (**B**) Connectivity maps for each study of PD-MCI were generated and overlaid, showing peak network overlap in the lateral temporal cortex. Section shown is at *z* = −18. (**C**) Direct comparison of network maps generated from studies of PD dementia and PD-MCI shows specificity of hippocampal connectivity to studies of PD dementia. Map is masked to the hippocampi and FWE-corrected *P* < 0.05. Section shown is at *z* = −14. (**D**) Connectivity to our a priori ROI in the hippocampus was significantly stronger for studies of PD dementia compared to studies of PD-MCI. (**E**) At lower network overlap thresholds, there are similarities between PD-MCI and PD dementia. This suggests that posterior nodes of the DMN are affected in both PD-MCI and PD dementia, and that at later stages, once PD dementia takes hold, hippocampal networks are affected. Sections shown are at *z* = 30, *z* = 21, *z* = −7 and *z* = −16. Coordinate and network maps for all studies can be viewed in Fig. 5. * *P* < 0.05; PDD, Parkinson’s disease dementia; PD-MCI, Parkinson’s disease with mild cognitive impairment.

**Figure 5:**
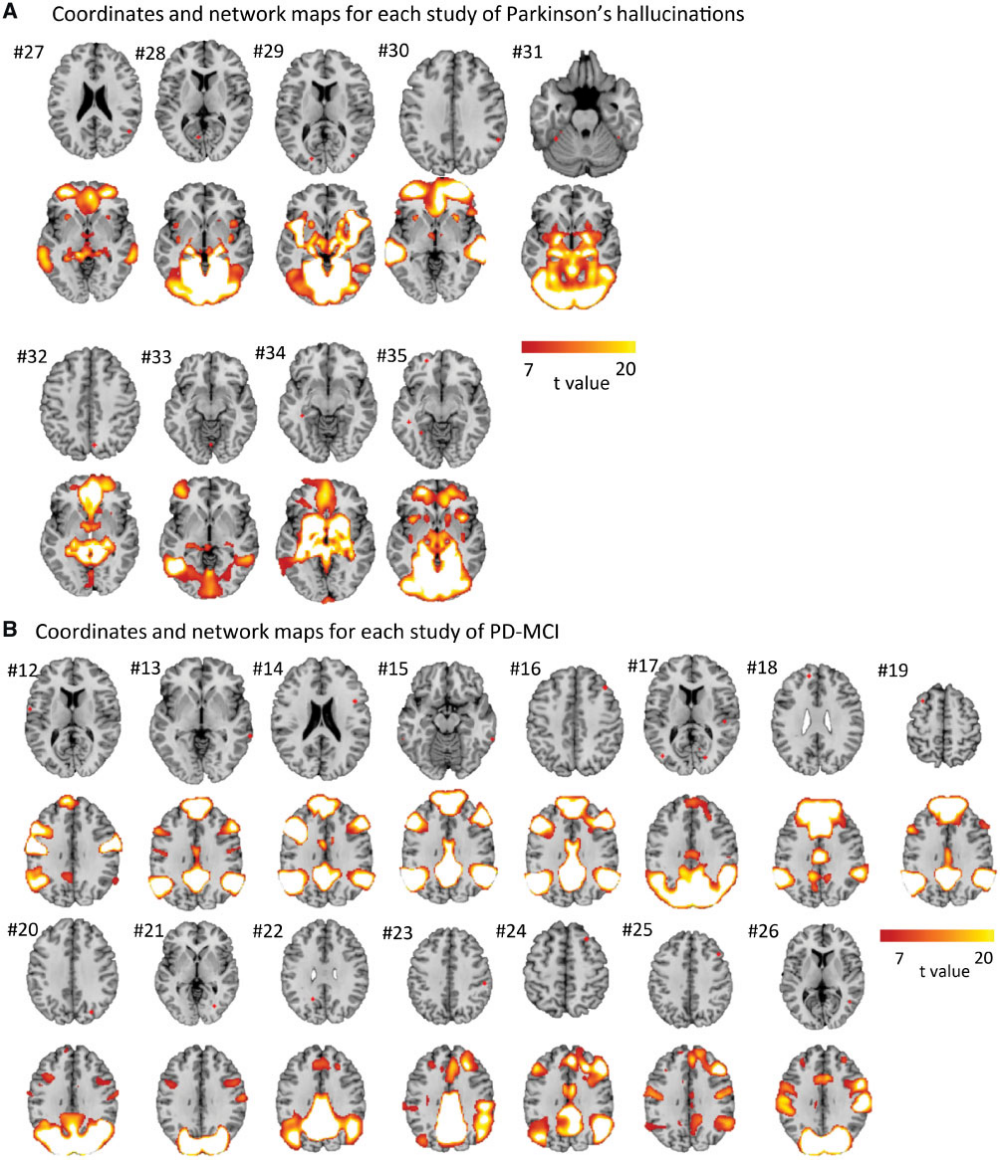
**Coordinate maps and network maps for Parkinson’s Hallucinations and for PD-MCI.** (**A**) Location of coordinates and of network connectivity for each study of Parkinson’s with hallucinations compared with Parkinson’s without hallucinations. (**B**) Location of coordinates and of network connectivity for each study of PD-MCI compared with Parkinson’s without cognitive involvement. Numbers refer to number of study in Tables 2 and 3.

Standard meta-analysis techniques again revealed heterogeneity of neuroimaging findings across studies (Figs 4A and 5), with no significant coordinates at either FDR or cluster inference levels of correction. Only 3/15 studies (20%) contributed to the most consistent clusters.

Using coordinate network mapping, over 80% of studies showed connectivity to posterior nodes of the default mode network (DMN), with peak overlap in lateral temporal cortex (Fig. 4B). Directly testing for specificity of hippocampal connectivity for PD dementia versus PD-MCI, we found that coordinates from studies of PD dementia were more connected to voxels in the right hippocampus (*P *<* *0.05, FWE-corrected, Fig. 4C) and more connected to our a priori hippocampal ROI (*z* = −2.2, *P *=* *0.013, one-tailed permutation test, Fig. 4D) compared to studies of PD-MCI.

Importantly, PD-MCI and PD dementia are a spectrum of cognitive involvement, and therefore, as well as finding differences between studies of PD-MCI and PD dementia in hippocampal regions, we would expect to see similarities in other networks. We therefore examined our network maps for PD dementia and PD-MCI at lower thresholds and found many similarities, with both PD dementia and PD-MCI showing network overlap in posterior nodes of the DMN (Fig. 4E). As such, while the peak network overlap was different, and significantly different in the hippocampus, at lower thresholds similar networks were apparent.

## Discussion

We show that neuroimaging findings in Parkinson’s dementia are heterogeneous across different studies, but are part of a common brain network centred on the hippocampi. This result was symptom-specific, as visual hallucinations mapped to a different network centred on the lateral geniculate nucleus. This finding was also stage-specific, as neuroimaging findings in PD-MCI mapped to a network centred on the lateral temporal cortex and posterior brain regions.

### Network localization of heterogeneous neuroimaging findings

Our finding that neuroimaging abnormalities in Parkinson’s dementia localize to a connected brain network, rather than one specific brain region, is consistent with an accumulating literature on network localization of neuropsychiatric symptoms (Boes *et al.*, 2015; Fox, 2018; Joutsa *et al.*, 2018*a*) and neurodegenerative diseases (Seeley *et al.*, 2009; Zhou *et al.*, 2012). Recently, we validated a new method for testing whether heterogeneous neuroimaging coordinates across different studies localize to a connected brain network (Darby *et al.*, 2019). The current study further validates and extends this method, showing specificity for highly co-morbid symptoms (PD dementia versus PD hallucinations) and specificity for disease stage (PD dementia versus PD-MCI). These results suggest that coordinate-based network mapping may help address a variety of neuroimaging questions that have proven difficult to address with conventional methods.

### Parkinson’s dementia and hippocampal networks

If heterogeneous neuroimaging findings in PD dementia were going to localize to any brain network, a network centred on the hippocampus makes sense. Clinically, memory is always affected in patients with PD dementia (Bronnick *et al.*, 2007; Muslimovic *et al.*, 2007; Reid *et al.*, 2011), and memory tests best distinguish PD dementia from Parkinson’s disease (Kiesmann *et al.*, 2013). Pathologically, the hippocampus shows more Lewy bodies (Harding and Halliday, 2001; Apaydin *et al.*, 2002; Arnold *et al.*, 2013; Hall *et al.*, 2014) reduced cholinergic activity (Hall *et al.*, 2014), and progressive volume loss in PD dementia (Hwang *et al.*, 2013; Pagonabarraga *et al.*, 2013; Zarei *et al.*, 2013; Rektorova *et al.*, 2014).

Despite this evidence, the importance of the hippocampus in PD dementia has been a source of debate (Emre *et al.*, 2007; Irwin *et al.*, 2012; Hall *et al.*, 2014; Aarsland *et al.*, 2017). In particular, neuroimaging studies have reported abnormalities in numerous brain regions outside the hippocampus (Burton *et al.*, 2004; Klein *et al.*, 2010; Song *et al.*, 2011; Melzer *et al.*, 2012). The current results help reconcile this debate, showing that these heterogeneous neuroimaging abnormalities are part of a common brain network, centred on the hippocampus.

### Parkinson’s dementia, Alzheimer’s disease and hippocampal networks

Our results implicating a hippocampal network in PD dementia aligns with other studies implicating a hippocampal network in Alzheimer’s dementia (Seeley *et al.*, 2009; Crossley *et al.*, 2014; Darby *et al.*, 2019). There are several possibilities for this convergence. One possibility is that the patients with PD dementia included in the above neuroimaging studies have co-morbid Alzheimer’s dementia, leading to a similar network localization. PD dementia is thought to be clinically and neuropathologically distinct from Alzheimer’s disease (Aarsland *et al.*, 2005; Farlow and Cummings, 2008; Irwin *et al.*, 2017). However, in later disease stages the amnestic component can be similar (Janvin *et al.*, 2006*b*; Bronnick *et al.*, 2007; Emre *et al.*, 2007), and PD dementia is characterized by amyloid and tau-related pathology in addition to alpha-synuclein (Compta *et al.*, 2011; Irwin *et al.*, 2017). As such this possibility cannot be excluded.

A second possibility, and the one we favour, is that PD dementia and Alzheimer’s disease are distinct disorders with distinct pathologies, but either pathology can involve the hippocampal network and cause dementia. In Alzheimer’s disease, hippocampal networks are affected early, leading to early amnestic symptoms followed by dementia. In contrast, Parkinson’s disease affects other networks first (especially posterior nodes of the DMN (Tessitore *et al.*, 2012; Hou *et al.*, 2016)), but once hippocampal networks are affected, the patient develops memory impairment and dementia. Our finding of higher specificity for the right hippocampus, which is linked with spatial rather than verbal memory (Ezzati *et al.*, 2016), would also be consistent with the higher propensity for spatial rather than verbal memory changes in PD dementia (Noe *et al.*, 2004).

Finally, it is important to consider whether our convergent localization in Alzheimer’s disease and PD dementia could be an artefact of our network mapping technique. Our technique will be biased towards identification of network hubs connected to the greatest number of other brain regions. However, this is unlikely to explain the current results as other nodes in the DMN are equally if not more connected to other brain regions compared to the hippocampus (Buckner *et al.*, 2009). Second, network localization to the hippocampus is specific to disorders of memory impairment, including Alzheimer’s disease and PD dementia. Neuroimaging coordinates from studies of other neurodegenerative diseases (Darby *et al.*, 2019), co-morbid symptoms in PD dementia such as visual hallucinations (Fig. 3), or even PD-MCI, an earlier stage of PD dementia which involves minimal memory impairment (Fig. 4), fail to show network overlap in the hippocampus.

### Network localization of Parkinson’s hallucinations to the lateral geniculate nuclei

Although initially included as a control for PD dementia, our findings in PD hallucinations are important in their own right. Heterogeneous neuroimaging abnormalities in PD hallucinations were part of a common network centred on the LGN. A central role of the LGN in visual hallucinations in PD has been hypothesized since the 1930s (de Morsier, 1936, 1938; Carter and Ffytche, 2015) and supported by more recent evidence (Diederich *et al.*, 2014). Intriguingly, our previous work on brainstem lesions causing visual hallucinations also implicated the LGN (Boes *et al.*, 2015), suggesting a common network localization for visual hallucinations independent of the underlying aetiology (stroke versus Parkinson’s disease).

A potential mechanistic model for the role of the LGN in hallucinations centres on two modes of signalling: a tonic mode, and a burst mode (Jones, 2009). During tonic mode, LGN cells are relatively depolarized and the LGN acts as a relay between retina and visual cortex. During burst mode, thalamic cells become hyperpolarized and are more likely to be enhanced by feedback from higher cortical regions. Intriguingly, this state occurs during drowsy inattentiveness, the same state linked with hallucinations (Llinas and Steriade, 2006).

Note that our results implicating the LGN do not preclude the involvement of other brain regions in PD hallucinations, such as the DMN (Shine *et al.*, 2014; Yao *et al.*, 2014; Shine *et al.*, 2015). In fact, one recent theory suggests that thalamic denervation to regions such as the LGN may reduce DMN inhibition resulting in visual hallucinations (Xuereb *et al.*, 1991; Ricciardi *et al.*, 2015; Onofrj *et al.*, 2017).

Although PD-associated visual hallucinations are associated with cognitive impairment (Barnes and David, 2001), almost all studies of PD hallucinations controlled for cognition, and across studies, cognition was not poorer in PD patients with visual hallucinations. Recent reports also reveal that hallucinations can be seen at earlier stages of PD in the absence of cognitive involvement (Pagonabarraga *et al.*, 2016), consistent with separate underlying processes or neuroanatomical substrates.

### Network localization reveals involvement of posterior nodes of the default mode network in Parkinson’s mild cognitive impairment

Our finding that neuroimaging abnormalities in PD-MCI localize to a network centred on posterior nodes of the DMN is consistent with clinical evidence that the earliest cognitive deficits in PD involve visuospatial processing (Williams-Gray *et al.*, 2013; Weil *et al.*, 2017). Lewy-related pathology in posterior brain regions increases the risk of dementia in PD (Toledo *et al.*, 2016), loss of connectivity in the DMN correlates with cognitive performance in Parkinson’s disease (Tessitore *et al.*, 2012; Karunanayaka *et al.*, 2016) and reduced DMN functional connectivity is seen in lateral temporal nodes as well as posterior brain regions in patients with PD-MCI (Hou *et al.*, 2016). Importantly, posterior DMN involvement was seen in both PD-MCI and PD dementia, consistent with the notion that these are part of the same spectrum of disease. Our findings are consistent with a model of cognitive impairment in PD that starts in posterior nodes of the DMN, causing early visuospatial deficits and MCI that eventually progresses to involve the hippocampal network as well, causing memory impairment and PD dementia. Such a model is consistent with the network propagation theory of neurodegenerative disease (Seeley *et al.*, 2009) and prion-like spread of alpha-synuclein (Zhou *et al.*, 2012).

Our findings are also consistent with the PD-related cognitive pattern identified by Eidelberg and colleagues using functional imaging (Huang *et al.*, 2007; Hirano *et al.*, 2012). This pattern of metabolic activity correlates with cognitive function in non-demented people with PD (Mattis *et al.*, 2011), with reduced activity in prefrontal and parietal regions. This pattern bears strong resemblance to the network we identified in PD-MCI (see Fig. 4).

### Limitations

There are several limitations to consider in our study. The numbers of studies included for each analysis are relatively small, particularly for PD dementia (*n* = 11) and Parkinson’s hallucinations (*n* = 9). These low study numbers undoubtedly contribute to the negative results of our activation likelihood estimation meta-analyses and those of other groups (Minkova *et al.*, 2017). Similarly, we used only one activation likelihood estimation meta-analysis technique. Other meta-analysis techniques have been applied in PD dementia and could produce different results (Pan *et al.*, 2013; Mihaescu *et al.*, 2018). Finally, due to these low study numbers, we used liberal one-tailed statistics for some analyses (e.g. connectivity of PD dementia coordinates versus PD hallucination coordinates to our a priori hippocampal ROI). We believe this was justified given clear a priori hypotheses regarding the direction of the finding; however, this result should be interpreted with caution until replicated. Note that the majority of our findings, including our voxel-wise analyses of hippocampal connectivity, were significant using standard two-tailed statistics. It is also important to note that studies with different numbers of coordinates did not dominate the analysis, as the coordinates for each study were used as a single (multi-location) seed.

A second limitation is clinical and study heterogeneity. For example, PD hallucinations can co-occur with cognitive impairment (Barnes and David, 2001) and PD-MCI can involve a range of cognitive domains (Litvan *et al.*, 2012). However, key clinical factors were controlled across studies: studies of PD dementia and PD-MCI controlled for motor impairment and disease stage, while studies of PD hallucinations controlled for cognition. Moreover, any heterogeneity across studies should bias us against the current findings of common network localization.

Finally, we used a normative connectome to link heterogeneous neuroimaging findings, similar to prior work from our lab (Darby *et al.*, 2018, 2019; Fox, 2018). However, one could argue that a connectome derived from Parkinson’s disease patients should work better for linking neuroimaging findings in Parkinson’s disease patients. Although intuitive, prior studies suggest that using disease-specific connectomes has minimal effect on network mapping results, and if anything weakens results due to worse signal to noise inherent in patient-based connectomes (Horn *et al.*, 2017; Weigand *et al.*, 2018).

## Funding

R.S.W. is supported by a Wellcome Clinical Research Career Development Fellowship (201567/Z/16/Z). M.D.F. is supported by funding from the Sidney R. Baer, Jr. Foundation, the NIH (R01MH113929), the Nancy Lurie Marks Foundation and the G. Harold and Leila Y. Mathers Charitable Foundation. R.R.D. is supported by funding from the Alzheimer’s foundation, Brightfocus foundation, Sidney Baer, Jr. Foundation and Vanderbilt faculty research scholars program.

## Competing interests

R.W. reports personal fees from General Electric.

## Abbreviations

ALE: activation likelihood estimation
DMN: default mode network
H&Y: Hoehn and Yahr
LGN: lateral geniculate nucleus
MCI: mild cognitive impairment
MMSE: mini-mental state examination
PD dementia: Parkinson’s disease with dementia
PD-PMCI: mild cognitive impairment associated with Parkinson’s disease
PDNC: Parkinson’s disease with normal cognition
PDVH: PD with visual hallucinations
